# Effects of music-based interventions on sleep quality in older adults: a systematic review and meta-analysis

**DOI:** 10.3389/fpsyt.2026.1761499

**Published:** 2026-02-24

**Authors:** Yupeng He, Yingcong Liu, Junlin He, Aoyi Li

**Affiliations:** 1 Wuhan Conservatory of Music, Wuhan, Hubei, China; 2 Hansei University, Gunpo-si, Republic of Korea

**Keywords:** cognitive function, meta-analysis, music-based interventions, neuropsychiatric symptoms, older adults, sleep quality, systematic review

## Abstract

**Objective:**

This study aims to systematically evaluate the effects of music-based interventions on sleep quality in older adults and to further explore its impact on depression, anxiety, and cognitive function.

**Methods:**

A comprehensive literature search was conducted in CNKI, Wanfang Data, VIP Database, China Biology Medicine disc (CBM), PubMed, Web of Science, the Cochrane Library, and Embase from database inception to November 2025. Randomized controlled trials (RCTs) investigating music therapy in older adults were included. Meta-analysis was performed using RevMan 5.4 and Stata 18.0 software. The primary outcome was sleep quality as assessed by the Pittsburgh Sleep Quality Index (PSQI), while secondary outcomes included the Geriatric Depression Scale (GDS), Self-Rating Anxiety Scale (SAS), and Mini-Mental State Examination (MMSE). Fixed- or random-effects models were applied according to heterogeneity, and subgroup analyses were conducted.

**Results:**

A total of 14 studies published in Chinese and English were finally included, involving 1,730 participants, with 863 in the intervention groups and 867 in the control groups. Meta-analysis showed that music-based interventions significantly improved sleep quality in older adults (PSQI: MD = −3.37, 95% CI: −4.24 to −2.50, *P* < 0.00001). Compared with control groups, music-based interventions significantly reduced depressive (GDS: MD = −3.94, 95% CI: −4.90 to −2.97, *P* < 0.00001) and anxiety symptoms (SAS: MD = −9.28, 95% CI: −18.21 to −0.35, *P* = 0.04), and improved cognitive function (MMSE: MD = 2.86, 95% CI: 1.40 to 4.13, *P* = 0.0001). The subgroup analyses indicated that interventions lasting 4–8 weeks, with session durations of 30–45 min and a frequency of seven sessions per week, were associated with greater improvements in sleep quality.

**Conclusions:**

Music-based interventions significantly improve sleep quality in older adults and has beneficial effects on depressive and anxiety symptoms as well as cognitive function. As a safe, non-invasive, and non-pharmacological intervention, music interventions may serve as an effective adjunct in the management of sleep and mental health in older adults.

**Systematic Review Registration:**

https://www.crd.york.ac.uk/prospero/, identifier CRD420251270027.

## 1 Introduction

Sleep disturbances are common health problems among older adults (1). With advancing age, age-related alterations in circadian rhythm regulation, changes in sleep architecture, and an increased burden of chronic diseases and polypharmacy make older adults more vulnerable to sleep-related problems, including difficulty initiating sleep, impaired sleep maintenance, and reduced sleep efficiency (2). Epidemiological studies have shown that approximately one-third to two-thirds of older adults experience varying degrees of sleep problems, and the prevalence increases further with advancing age (3, 4). Persistent poor sleep quality is not only closely associated with psychological disorders such as depression and anxiety but may also accelerate cognitive decline and increase the risks of falls, cardiovascular events, and mortality (5, 6). At present, management strategies for sleep disturbances in older adults mainly include pharmacological and non-pharmacological interventions (7). Although sedative–hypnotic medications can improve sleep symptoms in the short term, long-term use is associated with adverse effects such as drug dependence, tolerance, cognitive impairment, and an increased risk of falls, particularly in older populations (8). Therefore, identifying safe, effective, and sustainable non-pharmacological interventions has become an important focus in the management of sleep problems among older adults.

In recent years, non-invasive and low-cost interventions using music as a medium have received increasing attention in the fields of sleep and mental health (9, 10). Existing literature generally distinguishes two broad categories of such interventions: (1) therapist-led, goal-oriented clinical music therapy and (2) music-related interventions primarily based on music listening. For consistency, the present study adopts the term music-based interventions as an operational definition, and key intervention characteristics of each included study were systematically extracted to facilitate the interpretation of potential heterogeneity. Evidence suggests that music-based interventions may exert beneficial effects on sleep quality by enhancing emotional experiences, promoting relaxation responses, and optimizing pre-sleep behavioral patterns (11). In older adults in particular, poor sleep is closely associated with negative emotional states such as depression and anxiety and interacts with stress-related physiological arousal (6). Moreover, sleep impairment has been linked to cognitive decline and an increased risk of dementia, whereas improvements in sleep may provide foundational support for the maintenance of cognitive function (5, 12). Accordingly, music-based interventions may reduce arousal levels through emotional regulation and relaxation mechanisms to improve sleep and may indirectly influence emotional and cognitive outcomes (10, 13–15).

However, existing studies vary substantially in terms of study populations, intervention modalities, and outcome measures, and the overall evidence specific to older adults remains limited. More specifically, several key issues persist: (1) intervention protocols differ considerably, including receptive music therapy versus traditional Chinese five-tone music interventions as well as standalone interventions versus those combined with usual care, leaving the relative effectiveness of different intervention types unclear; (2) optimal intervention parameters, such as intervention duration, session length, and frequency, have not been clearly defined, which constrains further evaluation of intervention effects; and (3) whether intervention effects differ across populations, such as community-dwelling older adults and patients with Alzheimer’s disease, remains to be further explored. Accordingly, the present study conducted a systematic review and meta-analysis of randomized controlled trials to evaluate the effects of music-based interventions on sleep quality, depression, anxiety, and cognitive function in older adults. Subgroup analyses were further performed to explore the potential influence of intervention characteristics and population features on intervention effects, with the aim of providing evidence-based support for the application of music-based interventions in older populations.

## 2 Methods

### 2.1 Registration and protocol

This systematic review and meta-analysis was registered in the International Prospective Register of Systematic Reviews (PROSPERO) (registration no. CRD420251270027). The study was conducted according to a predefined protocol and reported in accordance with the Preferred Reporting Items for Systematic Reviews and Meta-Analyses (PRISMA) guidelines.

### 2.2 Search strategy

A comprehensive literature search was conducted in CNKI, Wanfang Data, VIP, China Biology Medicine (CBM), PubMed, Web of Science, the Cochrane Library, and Embase from inception to November 2025. Search strategies were developed using a combination of controlled vocabulary terms (e.g., MeSH and Emtree) and free-text keywords. Chinese search terms included “音乐治疗 (music therapy)”, “音乐干预 (music intervention)”, “音乐疗法 (music treatment)”, “五音疗法 (five-tone therapy)”, “睡眠障碍 (sleep disorder)”, “失眠 (insomnia)”, “不寐 (sleeplessness)”, “睡眠质量 (sleep quality)”, “老年/老年人 (older adults/elderly)”, “老年疾病 (geriatric disease)”, and “养老 (elder care)”. English search terms included “music”, “music therapy”, “music intervention”, “sleep quality”, “sleep wake disorders”, “aged”, and “elderly”. Only studies published in Chinese or English were considered. The detailed search strategies for each database are provided in the supplementary materials.

### 2.3 Inclusion and exclusion criteria

Inclusion criteria: Studies were included if they met the following criteria: (1) Participants: Older adults aged ≥60 years with sleep problems. No restrictions were placed on diagnostic status, medication use, or other individual characteristics. (2) Intervention: Participants in the experimental groups received music-based interventions. (3) Comparators: Control groups received non-music-based interventions, including usual sleep care (e.g., sleep-related health guidance, optimization of the sleep environment, reduction of nocturnal disturbances, and daytime activity arrangement), sleep hygiene education (health education focused on sleep hygiene), routine oncology care (e.g., preoperative and postoperative nursing care), or pharmacological interventions. (4) the study design was a randomized controlled trial (RCT). (5) The study was published in Chinese or English.

Exclusion criteria: Studies were excluded if they met any of the following criteria: (1) duplicate publications, (2) study protocols, reviews, theoretical articles, case reports, or conference abstracts, or (3) studies in which music-based intervention was combined with other interventions.

Outcome measures: The primary outcome was sleep quality as assessed using the Pittsburgh Sleep Quality Index (PSQI). Secondary outcomes included depressive symptoms measured by using the Geriatric Depression Scale (GDS), anxiety symptoms as assessed by the Self-Rating Anxiety Scale (SAS), and cognitive function evaluated by using the Mini-Mental State Examination (MMSE).

### 2.4 Data extraction

Duplicates were identified and manually removed using EndNote 21. Based on the predefined inclusion and exclusion criteria, titles and abstracts were first screened to exclude obviously irrelevant studies, followed by full-text review to determine final eligibility. Three reviewers independently extracted key information, including authors, year of publication, participants’ age and sex, sample size, intervention and control conditions, outcome measures, and intervention frequency. Any discrepancies were resolved through discussion and, if necessary, adjudicated by a fourth reviewer to ensure the accuracy of study selection.

### 2.5 Quality assessment

The methodological quality of the included studies was independently assessed by three reviewers using the Cochrane Risk of Bias Tool. The assessment covered seven domains: random sequence generation, allocation concealment, blinding of participants and personnel, blinding of outcome assessment, completeness of outcome data, selective reporting, and other potential sources of bias. Each domain was judged as “low risk,” “high risk,” or “unclear risk” of bias.

### 2.6 Data synthesis and analysis

Meta-analyses were conducted using RevMan version 5.4 and Stata version 18.0. Continuous outcomes were pooled using mean differences (MDs) with corresponding 95% confidence intervals (95% CIs), applying the inverse-variance method. Statistical heterogeneity was assessed using ***χ***² test and the ***I***² statistic. A random-effects model was applied when substantial heterogeneity was detected (***I***² > 50% or ***P* <** 0.10), with between-study variance (***τ***²) estimated using the DerSimonian–Laird method.

Regarding publication bias, funnel plots were generated and Egger’s regression test was performed when at least 10 studies were included. When the number of included studies was limited or heterogeneity was high, the results of publication bias assessments were interpreted with caution.

## 3 Results

### 3.1 Study selection

**Figure 1:**
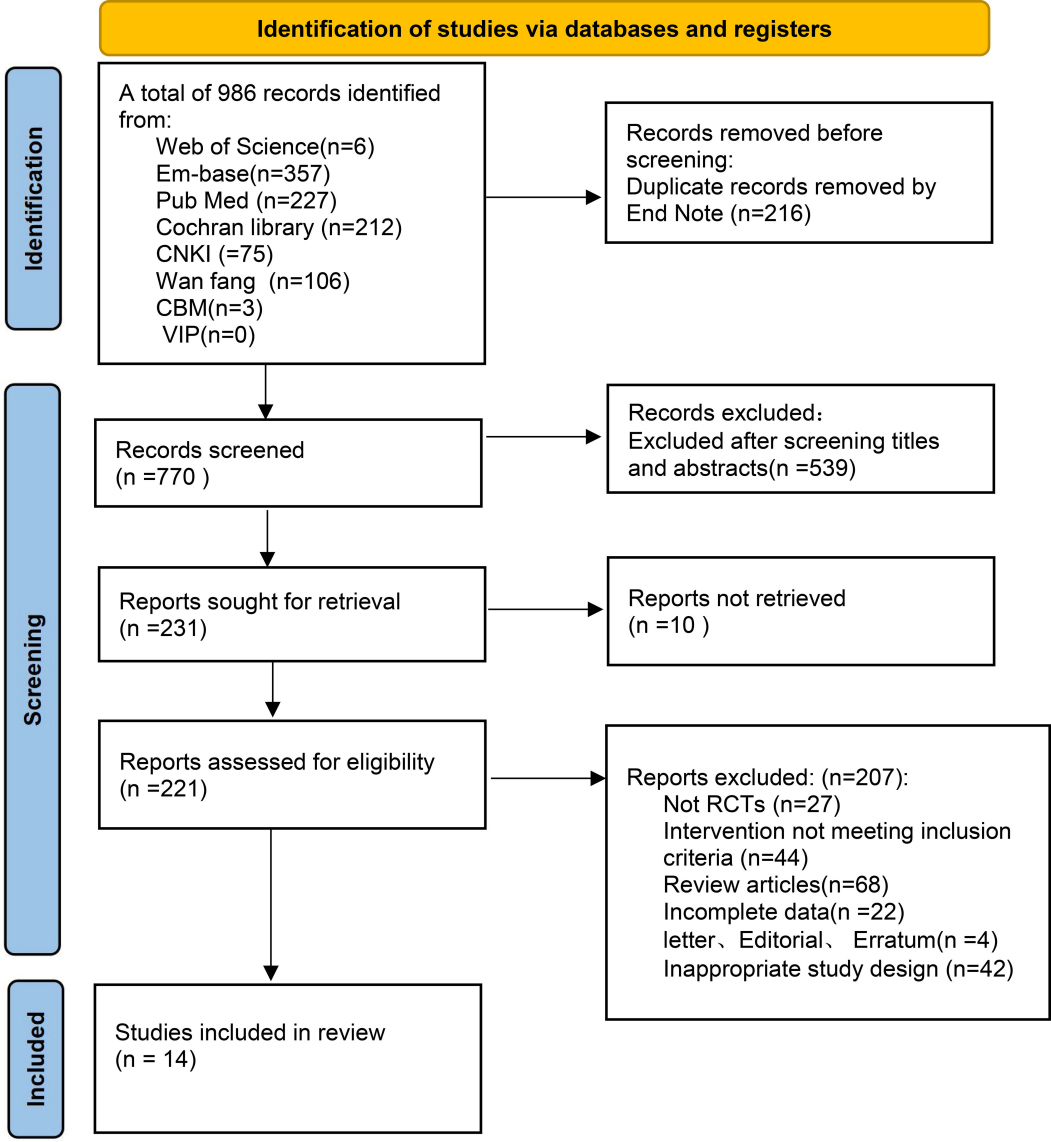
PRISMA flow diagram of study selection.

A total of 14 studies published in Chinese and English were finally included, involving 1,730 participants, with 863 in the intervention groups and 867 in the control groups. The literature screening and selection process is presented in **Figure 1**, and the main characteristics of the included studies are summarized in **Table 1**.

**Table 1:** Characteristics of the included studies.

Source	Group size/age		Intervention description		Intervention setting	Musical characteristics	Duration (weeks)	Session length (min)	Frequency (sessions/week)	Outcomes
	IG	CG	IG	CG						
Moon Fai Chan (16)	21/over 60	21/over 60	ML^a^	USC	Home environment	N/S	4	30	7	①
Jian Zhengqing (17)	23/65 ± 4	25/64 ± 4	ML^b^ + USC	USC	Home environment	N/S	4	30	N/S	①
Angela Shuma (18)	28/62.3 ± 6.3	32/65.4 ± 9.2	ML^a^	USC	Home environment	Sedative music	6	40	7	①
Qun Wang (19)	32/66.94 ± 4.99	32/69.82 ± 5.61	ML^a^ + SHE	SHE	Home environment	N/S	12	30–45	7	①
Sun Weiming (20)	48/67.16 ± 3.64	52/66.71 ± 3.56	GRMT^a^ + SHE	SHE	Therapy room	N/S	8	40	2	①
Dong Bo (21)	155/69.36 ± 6.34	155/69.36 ± 6.34	TCM^c^	PI + SHE	Therapy room	Five-element music	4	30	14	①
Li Yingchun (22)	60/69.22 ± 3.21	60/68.13 ± 3.56	GIM^c^	ROC	Hospital ward	N/S	4	55–65	5	①
Gao Yuting (23)	32/73.6 ± 2.4	32/73.4 ± 2.6	ML^c^ + USC	USC	Hospital ward	Marching and labor songs	12	30–60	28	①④
Shi Jin (24)	150/72.38 ± 2.07	150/71.98 ± 2.06	TCM^b^ + PI	PI	N/S	Five-element music	4	30	7	①
Wang Cong (25)	19/83.55 ± 6.0	19/81.80 ± 10.1	GRMT^a^	SHE	Therapy room	N/S	3	N/S	N/S	①④
Li Huiyuan (26)	46/81.76 ± 6.297	44/81.50 ± 7.679	ML^c^	SHE	Hospital ward	Nature-based relaxation music	8	60	N/S	①②③
Huang Shanggang (27)	36/75.42 ± 4.27	36/75.36 ± 4.36	TCM^c^	PI + USC	N/S	Five-element music	2	60	7	①
Shi Na (28)	42/67.42 ± 2.62	42/67.97 ± 2.36	ML^a^ + SHE	SHE	Hospital ward	N/S	4	30	7	①②③
Wang Xiaohui (29)	44/65.98 ± 5.49	44/65.98 ± 5.49	TCM^c^ + PI + USC	PI + USC	N/S	Five-element music	N/S	N/S	35	①

IG, intervention group; CG, control group; ML, music listening; GRMT, group receptive music therapy; TCM, traditional Chinese medicine five-tone music therapy; GIM, guided imagery and music; USC, usual sleep care; SHE, sleep hygiene education; ROC, routine oncology care; PI, pharmacological intervention; N/S, not specified; Five-element music, a genre of traditional Chinese music therapy based on the Wu Xing theory; ①, Pittsburgh Sleep Quality Index (PSQI); ②, Geriatric Depression Scale (GDS); ③, Self-Rating Anxiety Scale (SAS); ④, Mini-Mental State Examination (MMSE).

a Client-selected music (library).

b Unspecified music.

c Therapist-selected music.

### 3.2 Risk of bias assessment

**Figure 2:**
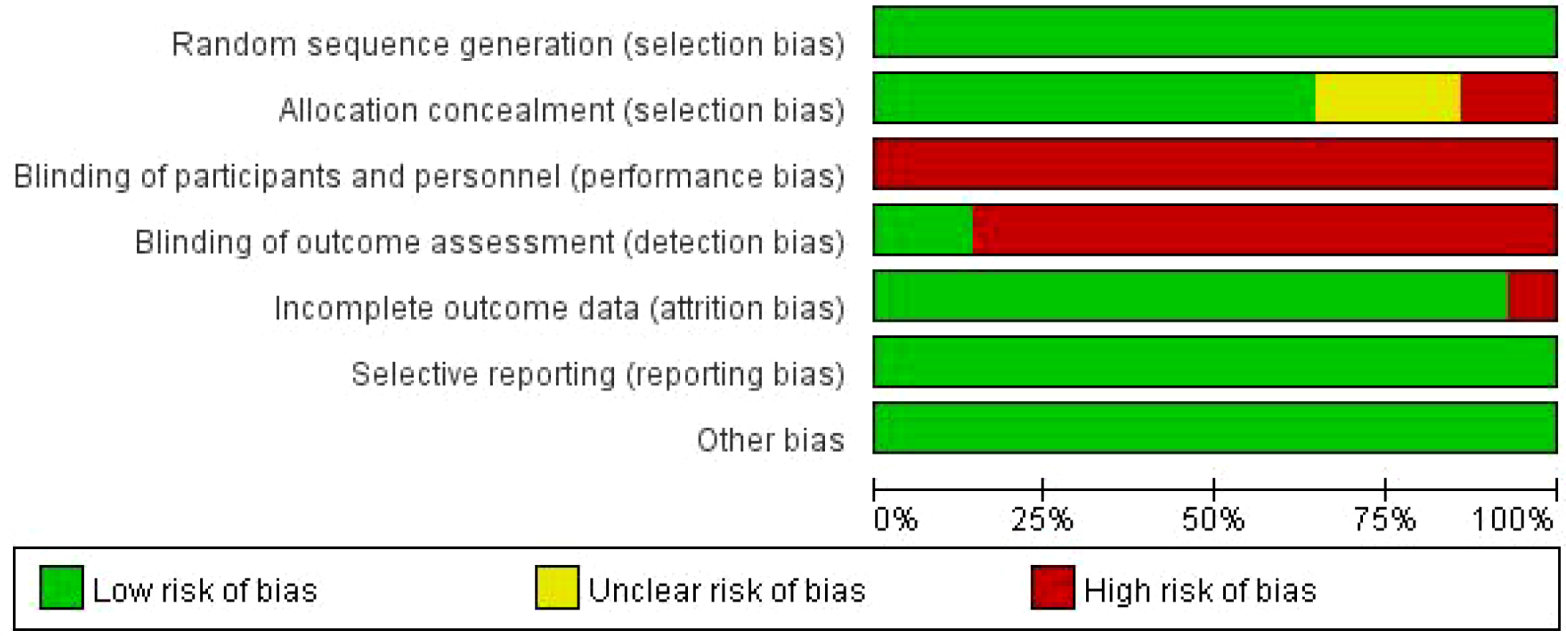
Risk of bias summary of the included studies.

**Figure 3:**
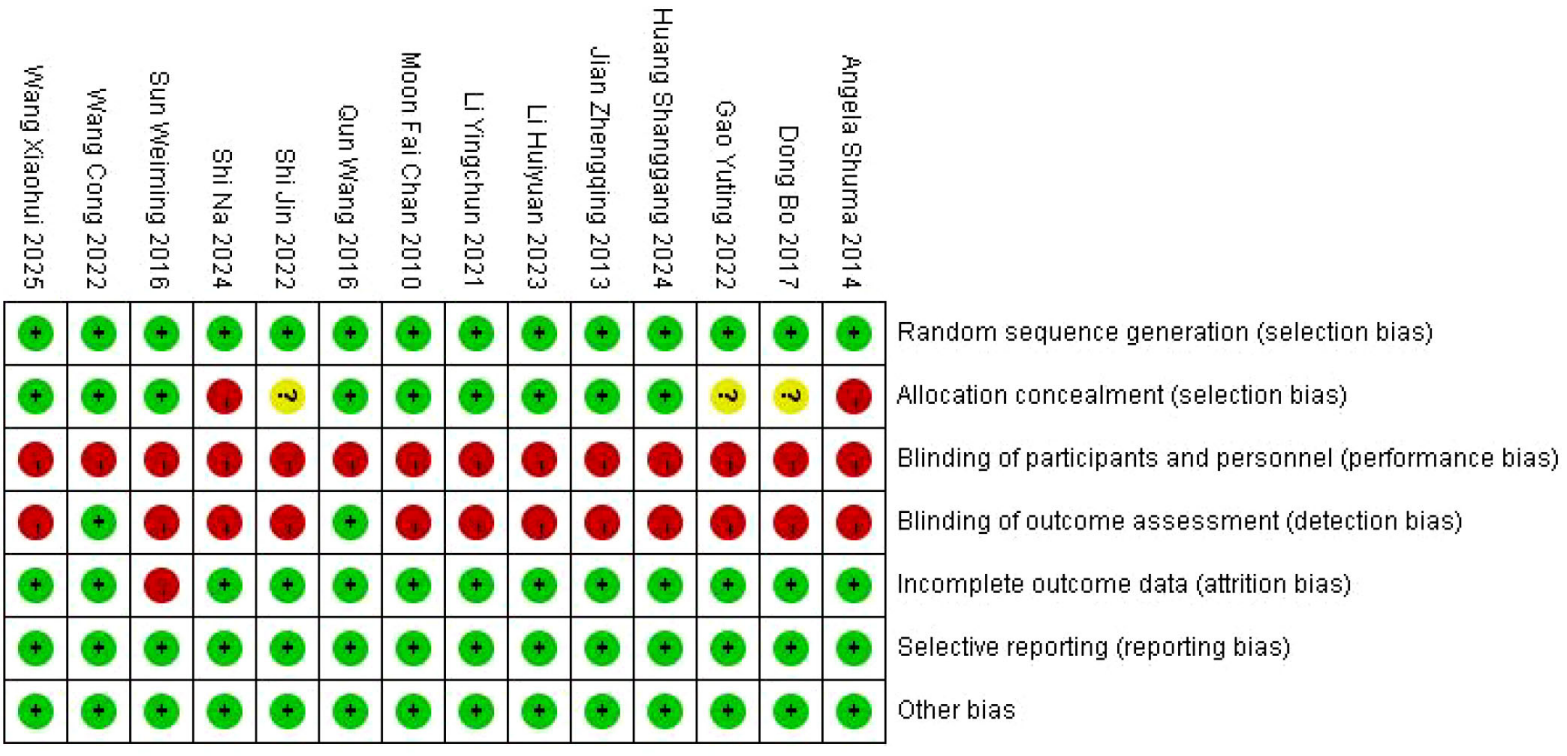
Risk of bias graph of the included studies.

The methodological quality of the included studies was assessed using the Cochrane Risk of Bias tool. Among the 14 included studies (**Figures 2**, **3**), nine reported appropriate randomization methods, including random number tables or computer-generated allocation. Only two studies (19, 25) reported blinding of outcome assessors, while blinding was not reported in the remaining studies. One study (20) reported participant attrition, whereas outcome data were complete in the other studies. No other sources of bias were identified.

### 3.3 Meta-analysis

#### 3.3.1 Effects of music-based interventions on sleep quality

**Figure 4:**
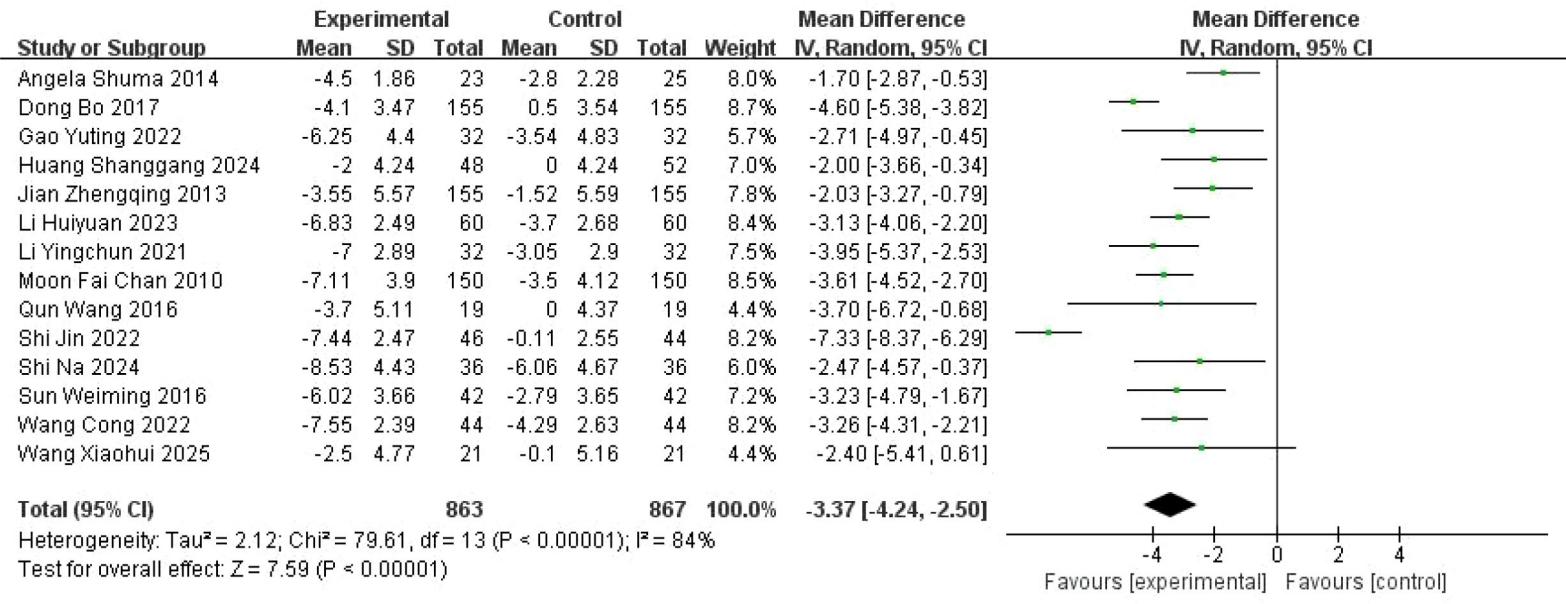
Forest plot of the effects of music-based interventions on sleep quality (PSQI).

**Figure 5:**
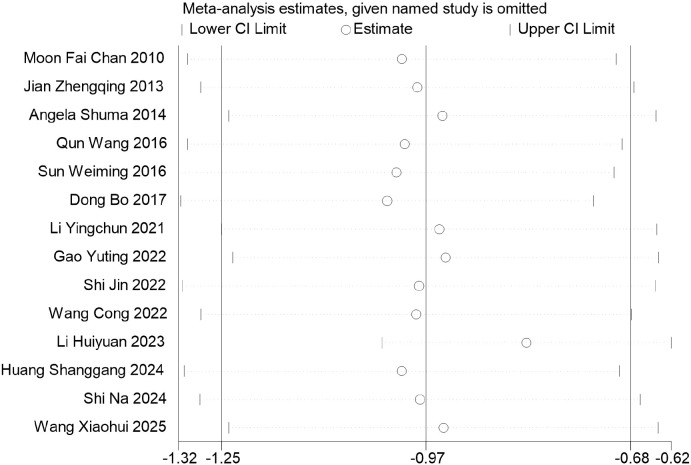
Sensitivity analysis for sleep quality (PSQI).

**Figure 6:**
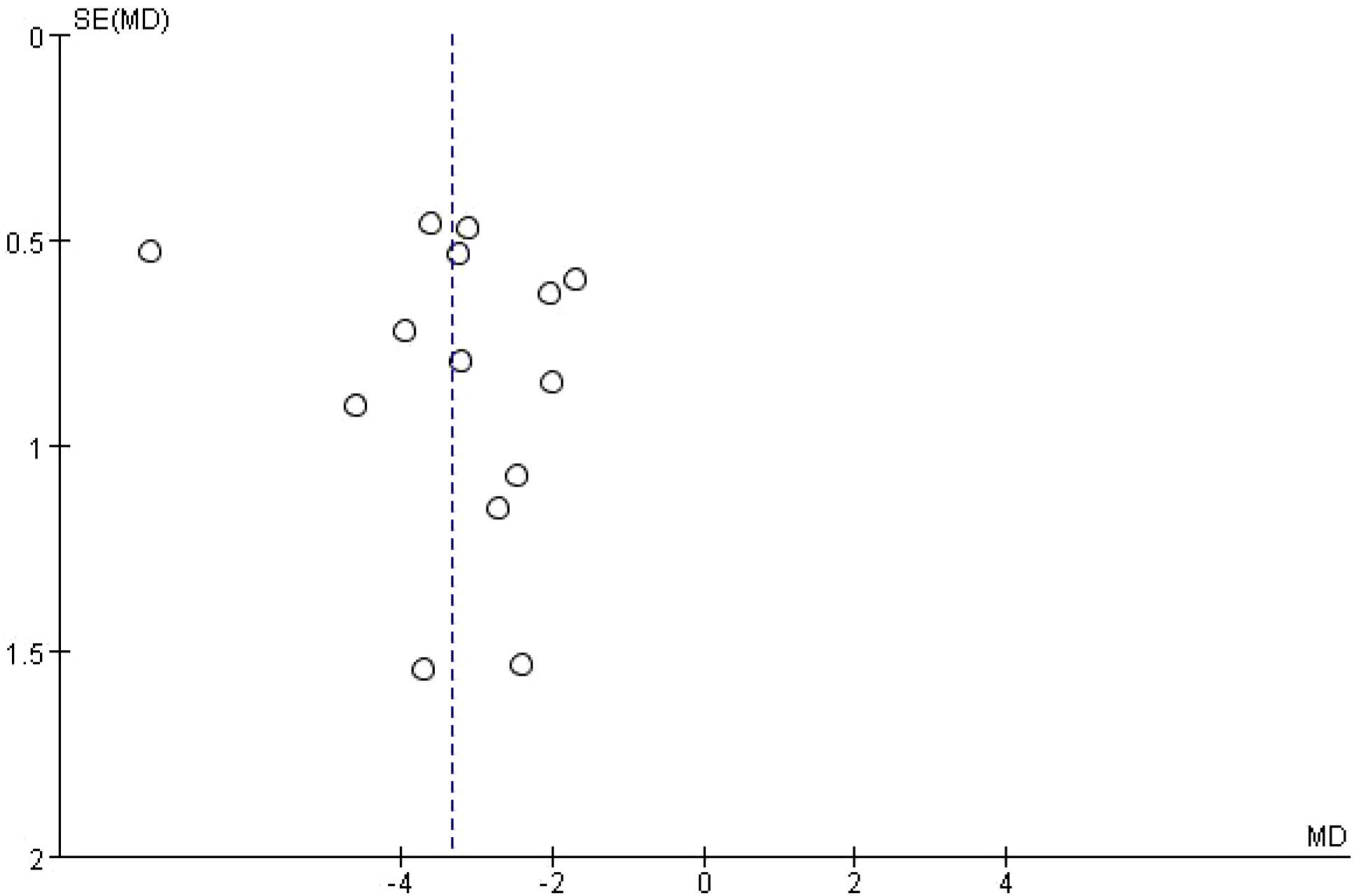
Funnel plot for publication bias of sleep quality outcomes.

A total of 14 studies were included to evaluate the effect of music-based interventions on sleep quality in older adults. The pooled analysis showed significant heterogeneity between studies (*I*² = 84%, *P* < 0.00001); thus, a random-effects model was applied (inverse-variance weighting), with between-study variance (*τ*²) estimated using the DerSimonian–Laird method (*τ*² = 2.28). The results showed that music-based interventions significantly improved sleep quality compared to the control group (MD = −3.37, 95% CI: −4.24 to −2.50, *P* < 0.00001) (**Figure 4**). A leave-one-out sensitivity analysis showed that the pooled mean difference (MD) ranged from −1.25 to −0.68, with consistent direction and statistical significance across all analyses (**Figure 5**). The funnel plot analysis (**Figure 6**) revealed no obvious asymmetry, and Egger’s regression test showed an intercept of −3.02 (95% CI: −6.88 to 0.84), *t*(12) = −1.70, and *P* = 0.114, indicating no significant small-study effects.

#### 3.3.2 Effects of music-based interventions on emotional outcomes

**Figure 7:**
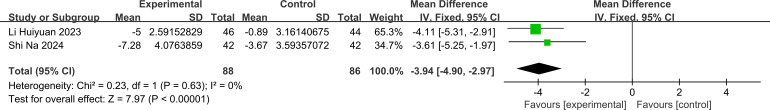
Forest plot of the effects of music-based interventions on depressive symptoms (GDS).

Two studies reported the effects of music-based interventions on depressive symptoms in older adults. No heterogeneity was observed between studies (*I*² = 0%, *P* = 0.63), so a fixed-effects model was applied. The results showed that the music-based interventions group significantly outperformed the control group in reducing Geriatric Depression Scale (GDS) scores (MD = −3.94, 95% CI: −4.90 to −2.97), with a statistically significant overall effect (*P* < 0.00001) (**Figure 7**).

**Figure 8:**
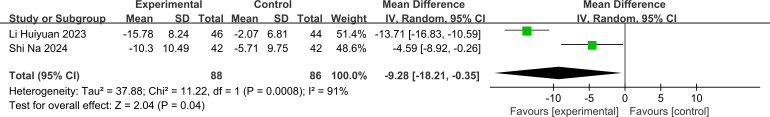
Forest plot of the effects of music-based interventions on anxiety symptoms (SAS).

Two studies reported the effects of music-based interventions on anxiety symptoms in older adults. Substantial heterogeneity was observed between studies (*I*² = 91%, *P* = 0.0008); therefore, a random-effects model was applied using inverse-variance weighting, with between-study variance (*τ*²) estimated using the DerSimonian–Laird method (*τ*² = 37.88). The pooled results showed that the music-based interventions group was significantly more effective than the control group in reducing Self-Rating Anxiety Scale (SAS) scores (MD = −9.28, 95% CI: −18.21 to −0.35), with a statistically significant overall effect (*P* = 0.04) (**Figure 8**).

#### 3.3.3 Effects of music-based interventions on cognitive function

**Figure 9:**
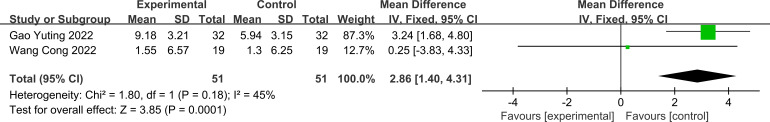
Forest plot of the effects of music-based interventions on cognitive function (MMSE).

Two studies reported the effects of music-based interventions on cognitive function in older adults, assessed using the Mini-Mental State Examination (MMSE). Heterogeneity between studies was low (*I*² = 45%, *P* = 0.18); therefore, a fixed-effects model was applied. The pooled results showed that, compared with the control group, the music-based interventions group achieved significantly higher MMSE scores (MD = 2.86, 95% CI: 1.40 to 4.13, *P* = 0.0001) (**Figure 9**).

### 3.4 Subgroup analysis

Subgroup analyses were conducted to explore potential sources of heterogeneity based on intervention duration, session length, intervention frequency, type of intervention, and target population (**Table 2**). Across all subgroups, music-based interventions were associated with improvements in sleep quality. Regarding intervention duration, interventions lasting 4–8 weeks showed the largest effect size (MD = −3.58, *P* < 0.00001). For session length, interventions lasting 30–45 min demonstrated the greatest improvement in sleep quality (MD = −3.60, *P* < 0.00001). In terms of intervention frequency, seven sessions per week were associated with the most pronounced effects (MD = −3.96, *P* < 0.00001). All intervention types showed significant effects on sleep quality. Improvements were more pronounced among community-dwelling older adults (MD = −2.99, *P* < 0.00001) compared with patients with Alzheimer’s disease.

**Table 2:** Subgroup analyses of the effects of music-based interventions on sleep quality (PSQI).

Subgroups	No. of studies	Total sample (I + C)	Heterogeneity		MD (95% CI)	Meta-analysis results	
			*I*(%)	*P*-value		*Z*	*P*
Duration (weeks)							
<4	2	110	37	0.21	-2.80 [-3.99, -1.61]	4.62	<0.00001
4–8	9	1,103	88	<0.00001	-3.58 [-4.81, -2.36]	5.73	<0.00001
>8	2	102	0	0.61	-3.07 [-4.88, -1.25]	3.32	0.0009
Session length (minutes)							
30–45	8	1,002	90	<0.00001	-3.60 [-5.13, -2.07]	4.62	<0.00001
>45	3	284	35	0.22	-3.11 [-4.03, -2.19]	6.62	<0.00001
Frequency (sessions/week)							
<7	2	220	0	0.5	-3.62 [-4.68, -2.57]	6.76	<0.00001
7	6	648	92	<0.00001	-3.96 [-4.49, -3.43]	14.64	<0.00001
>7	2	166	17	0.03	-3.62 [-4.88, -2.35]	5.59	<0.00001
Type of intervention							
ML	3	268	69	0.04	-2.87 [-3.91, -1.83]	5.42	<0.00001
ML + SC	4	484	0	0.77	-2.38 [-3.30, -1.46]	5.07	<0.00001
GRMT	2	172	0	0.98	-3.25 [-4.12, -2.38]	7.31	<0.00001
TCM	2	410	87	0.006	-3.41 [-5.95, -0.87]	2.63	0.009
TCM + SC	2	132	89	0.002	-5.08 [-9.89, -0.26]	2.97	0.04
Target population							
Patients with AD	3	416	0	0.87	-2.21 [-3.24, -1.19]	4.23	<0.0001
Community-dwelling older adults	5	354	41	0.15	-2.99 [-3.81, -2.18]	7.17	<0.00001

A negative MD indicates a greater reduction in PSQI scores (improved sleep) in the intervention group compared to the control group.

AD, Alzheimer’s disease; CI, confidence interval; MD, mean difference; SC, sleep care; ML, music listening; GRMT, group receptive music therapy; TCM, traditional Chinese medicine five-tone music therapy.

## 4 Discussion

This systematic review and meta-analysis synthesized evidence from 14 randomized controlled trials involving a total of 1,730 older adults to comprehensively evaluate the effects of music-based interventions on sleep outcomes in later life. The findings demonstrate that music-based interventions significantly improve sleep quality in older adults and exert additional beneficial effects on depressive symptoms, anxiety, and cognitive function. These results support the potential role of music therapy as an effective non-pharmacological intervention for sleep and mental health management in aging populations.

### 4.1 Effects and influencing factors of music-based interventions on sleep quality in older adults

Previous research has indicated that music-based interventions may influence sleep through multiple physiological and psychological pathways (13, 30). Listening to music characterized by slow tempo and gentle melodies before bedtime has been shown to reduce overall arousal levels and facilitate relaxation responses conducive to sleep initiation (31, 32). Objective polysomnographic studies further suggest that music interventions may decrease the proportion of light sleep (stage N1) and reduce high-frequency electroencephalographic activity, thereby promoting more stable sleep architecture (33). In addition, music can alleviate pre-sleep tension and rumination, redirecting attention away from stress-related cognitions and creating more favorable conditions for sleep onset (30).

On the basis of the mechanisms described above, the results of the present meta-analysis showed that music-based interventions significantly reduced the overall PSQI scores in older adults. The magnitude of improvement may be influenced by musical characteristics and intervention duration; specifically, sedative slow-tempo music and interventions lasting longer than 4 weeks appear to yield greater improvements in sleep quality. These findings are consistent with conclusions from previous systematic reviews and meta-analyses (34, 35). By including a larger number of randomized controlled trials and conducting subgroup analyses, the present study further confirmed the robustness of these findings and refined optimal intervention parameters.

The subgroup analyses revealed that music therapy improved sleep quality across different intervention durations, with the most pronounced effects observed in interventions lasting 4–8 weeks (MD = −3.58, *P* < 0.00001). This finding suggests that music therapy requires a sufficient intervention period to establish stable therapeutic effects. As a non-pharmacological approach, its benefits are typically achieved through repeated exposure, whereas excessively short interventions may be insufficient to induce sustained improvement, and overly prolonged interventions may reach a plateau due to habituation effects (11, 30, 32).

With regard to session length, interventions lasting 30–45 min demonstrated the greatest improvement in sleep quality (MD = −3.60, *P* < 0.00001), although beneficial effects were also observed with other durations. Moderate exposure time may help maintain engagement and relaxation, whereas longer sessions may induce fatigue or reduce adherence, thereby attenuating the intervention efficacy (10, 33).

In terms of intervention frequency, improvements in sleep quality were observed across studies employing regular music therapy, with the largest effect size associated with daily interventions (seven sessions per week; MD = −3.96, *P* < 0.00001). From a behavioral perspective, consistent and repeated musical exposure may strengthen the association between music listening and relaxation, facilitating the establishment of stable sleep–wake rhythms (11, 36).

The subgroup analyses by intervention modality indicated that multiple forms of music-based interventions significantly improved sleep quality in older adults (**Table 2**). This finding suggests that the therapeutic effects of music-based interventions may depend more on music-evoked emotional regulation and relaxation responses than on any specific musical form or theoretical framework (37). In addition, the subgroups in which music-based interventions were combined with usual care showed larger effect sizes in some comparisons, suggesting that multimodal approaches may provide synergistic benefits in sleep management for older adults.

Regarding population characteristics, beneficial effects were observed across different older adult groups, with relatively greater improvements among community-dwelling older adults (MD = −2.99, *P* < 0.00001). Previous studies suggest that baseline neurological status and disease burden may influence responsiveness to non-pharmacological interventions and that vulnerable populations may require longer or more individualized intervention strategies to achieve optimal outcomes (38, 39). Given the exploratory nature of the subgroup analyses, the limited number of studies in some subgroups, and the presence of heterogeneity, these findings should be interpreted with caution.

### 4.2 Effects of music-based interventions on emotional outcomes in older adults

Music has been shown to activate brain regions involved in emotional processing, including the prefrontal cortex, limbic system, and reward-related networks, thereby modulating affective responses and enhancing positive emotional experiences (13). Music listening may also influence the autonomic nervous system and the hypothalamic–pituitary–adrenal axis, reducing stress-related physiological arousal and alleviating anxiety (14, 15). Moreover, music-induced relaxation and emotional resonance can reduce negative rumination and enhance subjective emotion regulation. In older adults, familiar and personally meaningful music appears particularly effective in promoting emotional stability and alleviating depressive and anxiety symptoms (10, 38).

The present meta-analysis demonstrated that music-based interventions significantly reduced scores on both the Geriatric Depression Scale and the Self-Rating Anxiety Scale, indicating stable beneficial effects on depressive and anxiety symptoms in older adults. These findings are consistent with previous evidence supporting the role of music interventions in emotional regulation (15, 40). By including more diverse older adult samples and simultaneously examining both depressive and anxiety outcomes, the present study further reinforces the potential of music-based interventions as a non-pharmacological psychological intervention. Taken together, the results suggest that improvements in sleep quality may be accompanied by parallel benefits in emotional well-being.

### 4.3 Effects of music-based interventions on cognitive function in older adults

Music engagement activates widespread neural networks associated with cognitive processing and may support cognitive function through enhanced neuroplasticity (41). Neuroimaging studies have shown that music listening and music-based activities engage the prefrontal cortex, hippocampus, and default mode network, thereby supporting attention, memory, and executive functions (42). Regular musical engagement has also been proposed to help maintain functional connectivity within brain networks, which is particularly relevant in older adults and populations at risk of cognitive decline (43).

On this basis, the MMSE outcomes from the present meta-analysis suggest that music-based interventions may be associated with improved MMSE scores in older adults, consistent with findings from prior systematic reviews on music-based interventions and cognitive outcomes (41, 44). It should be noted, however, that the pooled MMSE analysis in the present study included two different intervention modalities—music listening and group receptive music therapy—resulting in a “mixed” estimate with limited clinical interpretability. Therefore, these findings should be interpreted with caution and are better considered as suggestive evidence. Given that improvements in sleep quality and reductions in emotional burden are closely linked to the maintenance of cognitive function (12, 45), future studies with consistent designs and clearly defined intervention modalities are needed to further clarify the independent contributions of different music-based interventions models to cognitive outcomes and to elucidate potential mechanisms.

## 5 Conclusions

The present study indicates that music-based interventions can significantly improve sleep quality in older adults and may have beneficial effects on depressive and anxiety symptoms as well as cognitive function. The subgroup analyses suggest that optimizing intervention parameters—particularly intervention duration, session length, and frequency—may enhance therapeutic effects. As a safe, non-invasive, and non-pharmacological approach, music-based interventions may serve as an effective adjunct in the management of sleep and mental health among older adults. Given differences in implementation settings and target populations, caution is warranted when extrapolating findings from community-based studies to institutional care environments. Future research should further examine the applicability of different music-based intervention modalities in institutional settings.

The present study also has several limitations, namely: (1) Some included studies provided insufficient information regarding randomization procedures and blinding, which may have affected the reliability of the findings. (2) Considerable variability existed across studies in terms of intervention modalities, music characteristics, intervention duration, and frequency, contributing to heterogeneity in the pooled effects. (3) The number of studies available for certain outcome measures was limited, and therefore the corresponding conclusions should be interpreted with caution. (4) Due to small sample sizes and differences in study design, findings from some subgroup analyses remain exploratory and require further confirmation.

## Data Availability

The original contributions presented in the study are included in the article/**Supplementary Material**. Further inquiries can be directed to the corresponding author.

## Supplementary material

The Supplementary Material for this article can be found onlineat: https://www.frontiersin.org/articles/10.3389/fpsyt.2026.1761499/full#supplementary-material

## References

[1] Miner B Kryger MH . Sleep in the aging population. Sleep Med Clin. (2017) 12:31–8. doi: 10.1016/j.jsmc.2016.10.008, PMID: 28159095 PMC5300306

[2] Crowley K . Sleep and sleep disorders in older adults. Neuropsychol Rev. (2011) 21:41–53. doi: 10.1007/s11065-010-9154-6, PMID: 21225347

[3] Ohayon MM Reynolds CF Dauvilliers Y . Excessive sleep duration and quality of life. Sleep Med. (2017) 33:13–21. doi: 10.1002/ana.23818, PMID: 23846792 PMC4142503

[4] Li J Vitiello MV Gooneratne NS . ). Sleep in normal aging. Sleep Med Clin. (2018) 13:1–11. doi: 10.1016/j.jsmc.2017.09.001, PMID: 29412976 PMC5841578

[5] Bubu OM Brannick M Mortimer J Umasabor-Bubu O Sebastião YV Wen Y . Sleep, cognitive impairment, and Alzheimer’s disease: A systematic review and meta-analysis. Sleep. (2017) 40:zsw032. doi: 10.1093/sleep/zsw032, PMID: 28364458

[6] Irwin MR . Why sleep is important for health: A psychoneuroimmunology perspective. Annu Rev Psychol. (2015) 66:143–72. doi: 10.1146/annurev-psych-010213-115205, PMID: 25061767 PMC4961463

[7] Riemann D Baglioni C Bassetti C Bjorvatn B Dolenc Groselj L Ellis JG . European guideline for the diagnosis and treatment of insomnia. J Sleep Res. (2017) 26:675–700. doi: 10.1111/jsr.12594, PMID: 28875581

[8] American Geriatrics Society Beers Criteria® Update Expert Panel . American Geriatrics Society 2019 updated AGS Beers Criteria^®^ for potentially inappropriate medication use in older adults. J Am Geriatr Soc. (2019) 67:674–94. doi: 10.1111/jgs.15767, PMID: 30693946

[9] Bradt J Dileo C Potvin N . Music for stress and anxiety reduction in coronary heart disease patients. Cochrane Database Syst Rev. (2016) 2016:CD006577. doi: 10.1002/14651858.CD006577.pub3, PMID: 24374731 PMC8454043

[10] Fancourt D Finn S . What is the evidence on the role of the arts in improving health and well-being? A scoping review. Copenhagen: WHO Regional Office for Europe (Health Evidence Network (HEN) synthesis report 67). (2019). 32091683

[11] Jespersen KV Koenig J Jennum P Vuust P . Music for insomnia in adults. Cochrane Database Syst Rev. (2015) 2015:CD010459. doi: 10.1002/14651858.CD010459.pub2, PMID: 26270746 PMC9241357

[12] Scullin MK Bliwise DL . Sleep, cognition, and normal aging: Integrating a half century of multidisciplinary research. Perspect Psychol Sci. (2015) 10:97–137. doi: 10.1177/1745691614556680, PMID: 25620997 PMC4302758

[13] Koelsch S . Investigating the neural encoding of emotion with music. Neuron. (2018) 98:1075–9. doi: 10.1016/j.neuron.2018.04.029, PMID: 29953870

[14] Linnemann A Ditzen B Strahler J Doerr JM Nater UM . Music listening as a means of stress reduction in daily life. Psychoneuroendocrinology. (2015) 60:82–90. doi: 10.1016/j.psyneuen.2015.06.008, PMID: 26142566

[15] de Witte M Spruit A van Hooren S Moonen X Stams GJJM . Effects of music interventions on stress-related outcomes: A systematic review and two meta-analyses. Health Psychol Rev. (2020) 14:294–324. doi: 10.1080/17437199.2019.1627897, PMID: 31167611

[16] Chan MF Chan EA Mok E . Effects of music on depression and sleep quality in elderly people: A randomised controlled trial. Complement Ther Med. (2010) 18:150–9. doi: 10.1016/j.ctim.2010.02.004, PMID: 20688261

[17] Jian ZQ Liu J Xia CH . Effects of positive music therapy on sleep disorders in older patients with dementia. Shanxi Med J. (2013) 42:1069–71.

[18] Shum A Taylor BJ Thayala J Chan MF . The effects of sedative music on sleep quality of older community-dwelling adults in Singapore. Complement Ther Med. (2014) 22:49–56. doi: 10.1016/j.ctim.2013.11.003, PMID: 24559816

[19] Wang Q Chai SY Wong EML Li X . The effects of music intervention on sleep quality in community-dwelling elderly. J Altern Complement Med. (2016) 22:576–84. doi: 10.1089/acm.2015.0304, PMID: 27223689

[20] Sun WM Yuan YF Dong XL Lu HL Zou Q He L . Effect of group music therapy on sleep quality in community-dwelling elderly. Chin J Gerontol. (2016) 36:6270–1. doi: 10.3969/j.issn.1005-9202.2016.24.6270

[21] Dong B Bu XM Zhang LJ Song YL Sun XT Liu M . Clinical effects of traditional Chinese five-tone music therapy on sleep disorders in older patients. Chin Gen Pract Nurs. (2017) 15:1204–5. doi: 10.3969/j.issn.1674-4748.2017.10.017

[22] Li YC Duan Y Tang SR Lang Y Zhao Y . Application of guided imagery music therapy to improve psychological distress and cancer-related sleep disorders in elderly patients with oral cancer. Chin. J. Convalescent Med. (2021) 30(12):1244–9. doi: 10.13517/j.cnki.ccm.2021.12.003

[23] Gao YT Qu Q . Effects of music intervention on sleep quality in patients with Alzheimer’s disease. Health Digest. (2022) 23:106–7.

[24] Shi J Qian W Zhang J . Integrated traditional Chinese and Western medical treatment and community nursing intervention for insomnia in older adults. Guide Chin Med Health Care. (2022) 12:101–4.

[25] Wang C . Effects of receptive music therapy on insomnia in older adults. ( Master’s thesis). Jilin University, China (2022).

[26] Li HY . Binaural beat music therapy for elderly people in nursing institutions: Effects of intervention on sleep and cognitive dysfunction. (Master's thesis). North China University of Science and Technology, Tangshan. (2023).

[27] Huang SG Liu MD Meng HQ Qin CM . To explore the application effect of TCM five-element music on elderly patients with mixed anxiety and depression disorder. Psychol. Mon. (2024) 19(16):141–3. doi: 10.19738/j.cnki.psy.2024.16.044

[28] Shi N Zhang XH Chen KY Lin XF . Effects of music therapy on hospitalized veteran older adults. Qilu J. Nurs. (2024) 30(19):81–4. doi: 10.3969/j.issn.1006-7256.2024.19.020

[29] Wang XH . Effects of TCM five-element music therapy on cognitive function and sleep quality in older adults with Alzheimer’s disease. World Latest Med Inf. (2025) 25(69):59–62. doi: 10.3969/j.issn.1671-3141.2025.69.018

[30] Dickson GT Schubert E . How does music aid sleep? literature review. Sleep Med. (2019) 63:142–50. doi: 10.1016/j.sleep.2019.05.016, PMID: 31655374

[31] Thoma MV La Marca R Brönnimann R Finkel L Ehlert U Nater UM . The effect of music on the human stress response. PloS One. (2013) 8:e70156. doi: 10.1371/journal.pone.0070156, PMID: 23940541 PMC3734071

[32] de Niet GJ Tiemens BG Lendemeijer HHGM Hutschemaekers GJM . Music-assisted relaxation to improve sleep quality: A meta-analysis. J Adv Nurs. (2009) 65:1356–64. doi: 10.1111/j.1365-2648.2009.04982.x, PMID: 19456998

[33] Cordi MJ Ackermann S Rasch B . Effects of relaxing music on healthy sleep. Sci Rep. (2019) 9:9079. doi: 10.1038/s41598-019-45608-0, PMID: 31235748 PMC6591240

[34] Chen CT Tung HH Fang CJ Wang YH Lin LC . Effect of music therapy on improving sleep quality in older adults: A systematic review and meta-analysis. J Am Geriatr Soc. (2021) 69:1925–32. doi: 10.1111/jgs.17149, PMID: 33880759

[35] Li C Kumar AP Wankhar D Kuppusamy M Govindasamy K . Effect of music therapy on sleep quality in elderly: A systematic review and meta-analysis. PLoS One. (2025) 20(11):e0334356. doi: 10.1371/journal.pone.0334356, PMID: 41187137 PMC12585030

[36] Harmat L Takács J Bódizs R . Music improves sleep quality in students. J Adv Nurs. (2008) 62:327–35. doi: 10.1111/j.1365-2648.2008.04602.x, PMID: 18426457

[37] Leubner D Hinterberger T . Reviewing the effectiveness of music interventions in treating depression. Front Psychol. (2017) 8:1109. doi: 10.3389/fpsyg.2017.01109, PMID: 28736539 PMC5500733

[38] Särkämö T . Music for the ageing brain: Cognitive, emotional, social, and neural benefits of musical leisure activities in stroke and dementia. Dementia (London). (2018) 17(6):670–85. doi: 10.1177/1471301217729237, PMID: 28895426

[39] Livingston G Huntley J Sommerlad A Ames D Ballard C Banerjee S . Dementia prevention, intervention, and care: 2020 report of the Lancet Commission. Lancet. (2020) 396:413–46. doi: 10.1016/S0140-6736(20)30367-6, PMID: 32738937 PMC7392084

[40] Aalbers S Fusar-Poli L Freeman RE Spreen M Ket JCF Vink AC . Music therapy for depression. Cochrane Database Syst Rev. (2017) 2017:CD004517. doi: 10.1002/14651858.CD004517.pub3, PMID: 29144545 PMC6486188

[41] Roman-Caballero R Arnedo M Triviño M Lupiáñez J . Musical practice as an enhancer of cognitive function in healthy aging: A systematic review and meta-analysis. PloS One. (2018) 13:e0207957. doi: 10.1371/journal.pone.0207957, PMID: 30481227 PMC6258526

[42] Jaschke AC Honing H Scherder EJA . Longitudinal analysis of music education on executive functions in primary school children. Front Neurosci. (2018) 12:103. doi: 10.3389/fnins.2018.00103, PMID: 29541017 PMC5835523

[43] Foster NEV Zatorre RJ . A role for the intraparietal sulcus in transforming musical pitch information. Cereb Cortex. (2010) 20(6):1350–9. doi: 10.1093/cercor/bhp199, PMID: 19789184

[44] Li HC Wang HH Chou FH Chen KM . The effect of music therapy on cognitive functioning among older adults: A systematic review and meta-analysis. J Am Med Dir Assoc. (2015) 16:71–7. doi: 10.1016/j.jamda.2014.10.004, PMID: 25458447

[45] Killgore WDS . Effects of sleep deprivation on cognition. Prog Brain Res. (2010) 185:105–29. doi: 10.1016/B978-0-444-53702-7.00007-5, PMID: 21075236

