# Supplementary material for: Effects of music-based interventions on sleep quality in older adults: a systematic review and meta-analysis
Source: Front Psychiatry. 2026 Feb 24;17:1761499. doi: 10.3389/fpsyt.2026.1761499 (PMC12971708; doi:10.3389/fpsyt.2026.1761499)
Supplement: Supplementary file 1 [file Supplementaryfile1.pdf]

Table S1 The search strategy

Datebases	Searching strategy	Literature number
Pubmed	("Music"[MeSH Terms] OR "Songs"[Title/Abstract] OR "Music"[Title/Abstract] OR "Song"[Title/Abstract] OR "vocal melody"[Title/Abstract] OR "melodies vocal"[Title/Abstract] OR "melody vocal"[Title/Abstract] OR "vocal melodies"[Title/Abstract] OR "classical music"[Title/Abstract] OR "music classical"[Title/Abstract] OR "jazz music"[Title/Abstract] OR "music jazz"[Title/Abstract] OR "rap music"[Title/Abstract] OR "music rap"[Title/Abstract] OR "hip hop music"[Title/Abstract] OR ("Hop"[All Fields] AND "music hip"[Title/Abstract]) OR "music hip hop"[Title/Abstract] OR ("Rock"[Title/Abstract] AND "roll music"[Title/Abstract]) OR "music therapy"[MeSH Terms] OR "music therapy"[Title/Abstract] OR "therapy music"[Title/Abstract]) AND ("Aged"[MeSH Terms] OR "Aged"[Title/Abstract] OR "Elderly"[Title/Abstract] OR "aged, 80 and over"[MeSH Terms] OR ("aged 80"[Title/Abstract] AND "over"[Title/Abstract]) OR "oldest old"[Title/Abstract]) AND ("sleep quality"[MeSH Terms] OR "sleep quality"[Title/Abstract] OR "qualities sleep"[Title/Abstract] OR "quality sleep"[Title/Abstract] OR "sleep wake disorders"[MeSH Terms] OR "disorder sleep wake"[Title/Abstract] OR "sleep wake disorders"[Title/Abstract] OR "disorder sleep wake"[Title/Abstract] OR "disorders sleep wake"[Title/Abstract] OR "sleep wake disorder"[Title/Abstract] OR "wake disorder sleep"[Title/Abstract] OR "wake disorders sleep"[Title/Abstract] OR "sleep disorders"[Title/Abstract] OR "disorder sleep"[Title/Abstract] OR "disorders sleep"[Title/Abstract] OR "sleep disorder"[Title/Abstract] OR ("Long"[All Fields] AND "sleeper syndrome"[Title/Abstract]) OR (("Long"[All Fields] AND ("Sleeper"[All Fields] OR "sleepers"[All Fields])) AND "Syndromes"[Title/Abstract]) OR (("Sleeper"[All Fields] OR "sleepers"[All Fields]) AND "syndrome long"[Title/Abstract]) OR (("Sleeper"[All Fields] OR "sleepers"[All Fields]) AND "syndromes long"[Title/Abstract]) OR (("syndrom"[All Fields] OR "syndromal"[All Fields] OR "syndromally"[All Fields] OR "Syndrome"[MeSH Terms] OR "Syndrome"[All Fields] OR "Syndromes"[All Fields] OR "syndrome s"[All Fields] OR "syndromic"[All Fields] OR "syndroms"[All Fields]) AND "long sleeper"[Title/Abstract]) OR (("syndrom"[All Fields] OR	227

	"syndromal"[All Fields] OR "syndromally"[All Fields] OR "Syndrome"[MeSH Terms] OR "Syndrome"[All Fields] OR "Syndromes"[All Fields] OR "syndrome s"[All Fields] OR "syndromic"[All Fields] OR "syndroms"[All Fields]) AND "long sleeper"[Title/Abstract]) OR "short sleeper syndrome"[Title/Abstract] OR ((("Short"[All Fields] OR "shorts"[All Fields]) AND ("Sleeper"[All Fields] OR "sleepers"[All Fields])) AND "Syndromes"[Title/Abstract]) OR (("Sleeper"[All Fields] OR "sleepers"[All Fields]) AND "syndrome short"[Title/Abstract]) OR (("Sleeper"[All Fields] OR "sleepers"[All Fields]) AND "syndromes short"[Title/Abstract]) OR (("syndrom"[All Fields] OR "syndromal"[All Fields] OR "syndromally"[All Fields] OR "Syndrome"[MeSH Terms] OR "Syndrome"[All Fields] OR "Syndromes"[All Fields] OR "syndrome s"[All Fields] OR "syndromic"[All Fields] OR "syndroms"[All Fields]) AND "short sleeper"[Title/Abstract]) OR (("syndrom"[All Fields] OR "syndromal"[All Fields] OR "syndromally"[All Fields] OR "Syndrome"[MeSH Terms] OR "Syndrome"[All Fields] OR "Syndromes"[All Fields] OR "syndrome s"[All Fields] OR "syndromic"[All Fields] OR "syndroms"[All Fields]) AND "short sleeper"[Title/Abstract]) OR "short sleep phenotype"[Title/Abstract] OR "phenotype short sleep"[Title/Abstract] OR "phenotypes short sleep"[Title/Abstract] OR "short sleep phenotypes"[Title/Abstract] OR "sleep phenotypes short"[Title/Abstract] OR "sleep related neurogenic tachypnea"[Title/Abstract] OR ((("Neurogenic"[All Fields] OR "neurogenically"[All Fields] OR "neurogenics"[All Fields]) AND ("Tachypnea"[MeSH Terms] OR "Tachypnea"[All Fields] OR "Tachypneas"[All Fields] OR "tachypnoea"[All Fields])) AND "Sleep-Related"[Title/Abstract]) OR ((("Neurogenic"[All Fields] OR "neurogenically"[All Fields] OR "neurogenics"[All Fields]) AND ("Tachypnea"[MeSH Terms] OR "Tachypnea"[All Fields] OR "Tachypneas"[All Fields] OR "tachypnoea"[All Fields])) AND "Sleep-Related"[Title/Abstract]) OR "sleep related neurogenic tachypnea"[Title/Abstract] OR (("Sleep-Related"[All Fields] AND ("Neurogenic"[All Fields] OR "neurogenically"[All Fields] OR "neurogenics"[All Fields])) AND "Tachypneas"[Title/Abstract]) OR (("Tachypnea"[MeSH Terms] OR "Tachypnea"[All Fields] OR "Tachypneas"[All Fields] OR "tachypnoea"[All Fields]) AND "sleep related neurogenic"[Title/Abstract]) OR (("Tachypnea"[MeSH Terms] OR "Tachypnea"[All Fields] OR "Tachypneas"[All Fields] OR "tachypnoea"[All Fields]) AND "sleep related neurogenic"[Title/Abstract]) OR ("Syndrome"[Title/Abstract]) OR ("Syndromes"[Title/Abstract]))	
Web of science	#1：TS=("Sleep Quality" OR "Qualities, Sleep" OR "Quality, Sleep" OR "Sleep Qualities" OR "Sleep Wake Disorders" OR "Disorder, Sleep Wake" OR "Disorders, Sleep Wake" OR "Sleep Wake Disorder" OR "Wake Disorder, Sleep" OR "Wake Disorders, Sleep" OR "Sleep Disorders" OR "Disorder, Sleep" OR "Disorders, Sleep" OR "Sleep Disorder" OR "Long Sleeper Syndrome" OR "Long Sleeper Syndromes" OR "Sleeper Syndrome, Long" OR "Sleeper Syndromes, Long"	**6**

	OR "Syndrome, Long Sleeper" OR "Syndromes, Long Sleeper" OR "Short Sleeper Syndrome" OR "Short Sleeper Syndromes" OR "Sleeper Syndrome, Short" OR "Sleeper Syndromes, Short" OR "Syndrome, Short Sleeper" OR "Syndromes, Short Sleeper" OR "Short Sleep Phenotype" OR "Phenotype, Short Sleep" OR "Phenotypes, Short Sleep" OR "Short Sleep Phenotypes" OR "Sleep Phenotypes, Short" OR "Sleep-Related Neurogenic Tachypnea" OR "Neurogenic Tachypnea, Sleep-Related" OR "Neurogenic Tachypneas, Sleep-Related" OR "Sleep Related Neurogenic Tachypnea" OR "Sleep-Related Neurogenic Tachypneas" OR "Tachypnea, Sleep-Related Neurogenic" OR "Tachypneas, Sleep-Related Neurogenic" OR "Subwakefullness Syndrome" OR "Subwakefullness Syndromes" OR "Syndromes, Subwakefullness" OR "Syndrome, Subwakefullness")<br><br>#2：TS=("Music" OR "Songs" OR "Song" OR "Vocal Melody" OR "Melodies, Vocal" OR "Melody, Vocal" OR "Vocal Melodies" OR "Classical Music" OR "Music, Classical" OR "Jazz Music" OR "Music, Jazz" OR "Rap Music" OR "Music, Rap" OR "Hip Hop Music" OR "Hop Music, Hip" OR "Music, Hip Hop" OR "Rock and Roll Music" OR "Music Therapy" OR "Therapy, Music" OR "Music Intervention" OR "Music Interventions" OR "Musical Intervention" OR "Musical Interventions" OR "Music-Based Intervention" OR "Music-Based Interventions")<br><br>#3：TS=("Aged" OR "Elderly" OR "Aged, 80 and over" OR "Oldest Old")<br><br>#4：#3 AND #2 AND #1	
Embase	#28 AND #75 AND #76 357<br><br>#34 OR #74 455989<br><br>#19 OR #24 57166	**357**

	#35 OR #36 OR #37 OR #38 OR #39 OR #40 OR #41 OR #42 OR #43 OR #44 OR #45 OR #46 OR #47 OR #48 OR #49 OR #50 OR #51 OR #52 OR #53 OR #54 OR #55 OR #56 OR #57 OR #58 OR #59 OR #60 OR #61 OR #62 OR #63 OR #64 OR #65 OR #66 OR #67 OR #68 OR #69 OR #70 OR #71 OR #72 OR #73	420818	
	'syndrome, subwakefullness':ab,ti	0	
	'syndromes, subwakefullness':ab,ti	0	
	'subwakefullness syndromes':ab,ti	0	
	'subwakefullness syndrome':ab,ti	0	
	'tachypneas, sleep-related neurogenic':ab,ti	0	
	'tachypnea, sleep-related neurogenic':ab,ti	0	
	'sleep-related neurogenic tachypneas':ab,ti	0	
	'sleep related neurogenic tachypnea':ab,ti	1	
	'neurogenic tachypneas, sleep-related':ab,ti	0	

	'neurogenic tachypnea, sleep-related':ab,ti	0	
	'sleep-related neurogenic tachypnea':ab,ti	1	
	'sleep phenotypes, short':ab,ti	1	
	'short sleep phenotypes':ab,ti	4	
	'phenotypes, short sleep':ab,ti	2	
	'phenotype, short sleep':ab,ti	2	
	'short sleep phenotype':ab,ti	30	
	'syndromes, short sleeper':ab,ti	0	
	'syndrome, short sleeper':ab,ti	0	
	'sleeper syndromes, short':ab,ti	0	
	'sleeper syndrome, short':ab,ti	0	
	'short sleeper syndromes':ab,ti	0	
	'short sleeper syndrome':ab,ti	2	

	'syndromes, long sleeper':ab,ti	0	
	'syndrome, long sleeper':ab,ti	0	
	'sleeper syndromes, long':ab,ti	0	
	'sleeper syndrome, long':ab,ti	0	
	'long sleeper syndromes':ab,ti	0	
	'long sleeper syndrome':ab,ti	0	
	'sleep disorder':ab,ti	10788	
	'disorders, sleep':ab,ti	1112	
	'disorder, sleep':ab,ti	457	
	'sleep disorders':ab,ti	32497	
	'wake disorders, sleep':ab,ti	10	
	'wake disorder, sleep':ab,ti	1	
	'sleep wake disorder':ab,ti	267	

	'disorders, sleep wake':ab,ti	25	
	'disorder, sleep wake':ab,ti	9	
	'sleep wake disorders':ab,ti	639	
	'sleep wake disorders'/exp	416345	
	#29 OR #30 OR #31 OR #32 OR #33	76861	
	'sleep qualities':ab,ti	85	
	'quality, sleep':ab,ti	3131	
	'qualities, sleep':ab,ti	4	
	'sleep quality':ab,ti	57223	
	'sleep quality'/exp	60234	
	#25 OR #26 OR #27	555894<br>3	
	'aged':ab,ti	125203<br>0	

	'elderly':ab,ti	474522	
	'aged'/exp	4631005	
	#20 OR #21 OR #22 OR #23	12899	
	'therapy, music':ab,ti	279	
	'music intervention':ab,ti	1067	
	'music therapy':ab,ti	6472	
	'music therapy'/exp	11933	
	#1 OR #2 OR #3 OR #4 OR #5 OR #6 OR #7 OR #8 OR #9 OR #10 OR #11 OR #12 OR #13 OR #14 OR #15 OR #16 OR #17 OR #18	54371	
	'rock and roll music':ab,ti	19	
	'music, hip hop':ab,ti	1	
	'hop music, hip':ab,ti	0	

	'hip hop music':ab,ti	36	
	'music, rap':ab,ti	8	
	'rap music':ab,ti	165	
	'music, jazz':ab,ti	8	
	'jazz music':ab,ti	215	
	'music, classical':ab,ti	53	
	'classical music':ab,ti	1415	
	'vocal melodies':ab,ti	12	
	'melody, vocal':ab,ti	3	
	'melodies, vocal':ab,ti	0	
	'vocal melody':ab,ti	5	
	'song':ab,ti	11545	
	'songs':ab,ti	3866	

Cochrane Library	#1 (Music):ti,ab,kw OR (Songs):ti,ab,kw OR (Song):ti,ab,kw OR (Vocal Melody):ti,ab,kw OR (Melodies, Vocal):ti,ab,kw 9312<br><br>#2 (Melody, Vocal):ti,ab,kw OR (Vocal Melodies):ti,ab,kw OR (Classical Music):ti,ab,kw OR (Music, Classical):ti,ab,kw OR (Jazz Music):ti,ab,kw 484<br><br>#3 (Music, Jazz):ti,ab,kw OR (Rap Music):ti,ab,kw OR (Music, Rap):ti,ab,kw OR (Hip Hop Music):ti,ab,kw OR (Hop Music, Hip):ti,ab,kw 71<br><br>#4 (Music, Hip Hop):ti,ab,kw OR (Rock and Roll Music):ti,ab,kw OR (Music Therapy):ti,ab,kw OR (Therapy, Music):ti,ab,kw 5079<br><br>#5 #1 OR #2 OR #3 OR #4 9312<br><br>#6 (Aged):ti,ab,kw OR (Elderly):ti,ab,kw OR (Aged, 80 and over):ti,ab,kw OR (Oldest Old):ti,ab,kw 744576<br><br>#7 (Sleep Quality):ti,ab,kw OR (Qualities, Sleep):ti,ab,kw OR (Quality, Sleep):ti,ab,kw OR (Sleep Qualities):ti,ab,kw OR ("sleep/wake disorders"):ti,ab,kw 30630<br><br>#8 (Disorder, Sleep Wake):ti,ab,kw OR (Disorders, Sleep Wake):ti,ab,kw OR (Sleep Wake Disorder):ti,ab,kw OR (Wake Disorder, Sleep):ti,ab,kw OR (Wake Disorders, Sleep):ti,ab,kw 4368<br><br>#9 (Sleep Disorders):ti,ab,kw OR (Disorder, Sleep):ti,ab,kw OR (Disorders, Sleep):ti,ab,kw OR (Sleep Disorder):ti,ab,kw OR (Long Sleeper Syndrome):ti,ab,kw 23432<br><br>#10 (Long Sleeper Syndromes):ti,ab,kw OR (Sleeper Syndrome, Long):ti,ab,kw OR (Sleeper Syndromes, Long):ti,ab,kw OR (Syndrome, Long Sleeper):ti,ab,kw OR (Syndromes, Long Sleeper):ti,ab,kw 4<br><br>#11 (Syndromes, Short Sleeper):ti,ab,kw OR (Short Sleep Phenotype):ti,ab,kw OR (Phenotype, Short Sleep):ti,ab,kw OR (Phenotypes, Short Sleep):ti,ab,kw OR (Short Sleep Phenotypes):ti,ab,kw 73	**212**

	#12 (Short Sleeper Syndrome):ti,ab,kw OR (Short Sleeper Syndromes):ti,ab,kw OR (Sleeper Syndrome, Short):ti,ab,kw OR (Sleeper Syndromes, Short):ti,ab,kw OR (Syndrome, Short Sleeper):ti,ab,kw 3<br><br>#13 (Sleep Phenotypes, Short):ti,ab,kw OR (Sleep-Related Neurogenic Tachypnea):ti,ab,kw OR (Neurogenic Tachypnea, Sleep-Related):ti,ab,kw OR (Neurogenic Tachypneas, Sleep-Related):ti,ab,kw OR (Sleep Related Neurogenic Tachypnea):ti,ab,kw 30<br><br>#14 (Sleep-Related Neurogenic Tachypneas):ti,ab,kw OR (Tachypnea, Sleep-Related Neurogenic):ti,ab,kw OR (Tachypneas, Sleep-Related Neurogenic):ti,ab,kw OR (Subwakefullness Syndrome):ti,ab,kw OR (Subwakefullness Syndromes):ti,ab,kw 0<br><br>#15 (Syndromes, Subwakefullness):ti,ab,kw OR (Syndrome, Subwakefullness):ti,ab,kw 0<br><br>#16 #7 OR #8 OR #9 OR #10 OR #11 OR #12 OR #13 OR #14 OR #15 41372<br><br>#17 #5 AND #6 AND #16 212<br><br>#18 (Music Intervention):ti,ab,kw 5036<br><br>#19 #5 OR #18 9312<br><br>#20 #19 AND #6 AND #16 212	
CNKI	SU=('音乐治疗' + '音乐干预' + '音乐疗法' + '五音疗法') and SU=('睡眠障碍' + '睡眠异常' + '睡眠困难' + '失眠' + '不寐' + '睡眠质量') and SU=('老年' + '老年人' + '老年疾病' + '养老')	**75**

Wanfang Data	(主题:("音乐治疗" OR "音乐干预" OR "音乐疗法" OR "五音疗法")) AND (主题:("睡眠障碍" OR "睡眠异常" OR "睡眠困难" OR "失眠" OR "不寐" OR "睡眠质量")) AND (主题:("老年" OR "老年人" OR "老年疾病" OR "养老"))	**106**
CBM	((主题=音乐*) AND 主题=老年*) AND 主题=睡眠*	**3**
VIP Database	(M=('音乐治疗' + '音乐干预' + '音乐疗法' + '五音疗法')) * (M=('睡眠障碍' + '睡眠异常' + '睡眠困难' + '失眠' + '不寐' + '睡眠质量')) * (M=('老年' + '老年人' + '老年疾病' + '养老'))	**0**
